# Authors’ Reply: Enhancing Patient Support in Digital Inflammatory Bowel Disease Tools: The Need for Medication Guidance and Decision Support

**DOI:** 10.2196/83160

**Published:** 2025-09-26

**Authors:** Kaitlyn Chappell, Karen Wong

**Affiliations:** 1 Division of Gastroenterology, Department of Medicine, University of Alberta, 130 University Campus NW, Edmonton, AB, Canada, 1 7804928691 ext 6

**Keywords:** digital health, self-management, electronic medical records, quality of life, diet, mental health, inflammatory bowel disease, social determinants of health, implementation

Thank you for your thoughtful letter regarding our protocol for the MyIBDToolkit study [1,2]. We appreciate your insights from both a clinical and a patient advocacy perspective, which align with our core goal of creating a patient-centered tool that addresses the complexities of inflammatory bowel disease (IBD) care.

We agree that providing comprehensive, patient-specific medication guidance and responsive symptom-based support is crucial for effective self-management. MyIBDToolkit is designed to incorporate these features. The patient-facing tools in our protocol include questionnaires and flowsheets for tracking symptoms and a flare questionnaire that collects patient-reported data to inform responsive decision support. We recognize that decision support tools may help health care provider (HCP) adherence to protocols [3]. Still, we plan to evaluate how HCPs and patients use these tools before exploring the addition of more standardized decision support.

A key challenge with IBD medications is that they are highly individualized for each patient [4]. We have chosen to empower HCPs to manage medication messaging directly. With our tool, doctors can access and customize “smart phrases,” or prewritten text templates, to provide precise instructions [5]. To balance the evolving nature of treatments with our capacity to update information, we’ve created a linked information page on our provincial health website [6] that provides information on IBD medications and will be updated regularly under the oversight of a team of HCPs. The toolkit also includes mini self-management pathways that offer patients vetted resources for mental health and nutrition. We believe these will provide the self-care suggestions you recommend.

The HCP-targeted tools we are building, such as the “IBD Overview” and “IBD Navigator,” are designed to standardize care and facilitate comanagement. This will ensure medication information is consistently recorded and visible to patients on their portal, which we believe may help reduce the information overload patients often feel after appointments.

We trust the feedback from our patient advisory council and physician advisory board to address any future gaps. We continually use their input to refine the MyIBDToolkit. The tiered, phased rollout of our study is designed to collect and analyze this feedback, informing subsequent iterations. We also acknowledge that building within our province’s existing electronic health record system limits our ability to add certain features.

We are confident that by working closely with our patient partners and continuously improving the tool based on implementation metrics, we can create a comprehensive resource that improves health care use and empowers patients with the confidence to manage their complex condition.

Thank you again for your valuable feedback. It strengthens our resolve to create a truly impactful digital health solution for the IBD community.

## Abbreviations

HCP: health care provider
IBD: inflammatory bowel disease
